# Comparative oncology DNA sequencing of canine T cell lymphoma via human hotspot panel

**DOI:** 10.18632/oncotarget.25209

**Published:** 2018-04-27

**Authors:** J. Tyson McDonald, Athena Kritharis, Afshin Beheshti, Monika Pilichowska, Kristine Burgess, Luisel Ricks-Santi, Elizabeth McNiel, Cheryl B. London, Dashnamoorthy Ravi, Andrew M. Evens

**Affiliations:** ^1^ Cancer Research Center, Hampton University, Hampton, VA, USA; ^2^ Division of Blood Disorders, Rutgers Cancer Institute of New Jersey, New Brunswick, NJ, USA; ^3^ WYLE, NASA Ames Research Center, Moffett Field, CA, USA; ^4^ Division of Pathology, Tufts Medical Center, Boston, MA, USA; ^5^ Cummings School of Veterinary Medicine, Tufts University, Boston, MA, USA

**Keywords:** canine, T cell lymphoma, DNA sequencing, cancer hotspots, Non-Hodgkin lymphoma

## Abstract

T-cell lymphoma (TCL) is an uncommon and aggressive form of human cancer. Lymphoma is the most common hematopoietic tumor in canines (companion animals), with TCL representing approximately 30% of diagnoses. Collectively, the canine is an appealing model for cancer research given the spontaneous occurrence of cancer, intact immune system, and phytogenetic proximity to humans. We sought to establish mutational congruence of the canine with known human TCL mutations in order to identify potential actionable oncogenic pathways. Following pathologic confirmation, DNA was sequenced in 16 canine TCL (cTCL) cases using a custom Human Cancer Hotspot Panel of 68 genes commonly mutated in human TCL. Sequencing identified 4,527,638 total reads with average length of 229 bases containing 346 unique variants and 1,474 total variants; each sample had an average of 92 variants. Among these, there were 258 germline and 32 somatic variants. Among the 32 somatic variants there were 8 missense variants, 1 splice junction variant and the remaining were intron or synonymous variants. A frequency of 4 somatic mutations per sample were noted with >7 mutations detected in *MET*, *KDR*, *STK11* and *BRAF*. Expression of these associated proteins were also detected via Western blot analyses. In addition, Sanger sequencing confirmed three variants of high quality (*MYC*, *MET*, and *TP53* missense mutation). Taken together, the mutational spectrum and protein analyses showed mutations in signaling pathways similar to human TCL and also identified novel mutations that may serve as drug targets as well as potential biomarkers.

## INTRODUCTION

Peripheral T-cell lymphomas (PTCLs) are an uncommon and heterogeneous group of cancers constituting approximately 10–15% of all human non-Hodgkin lymphomas (NHLs) in Western countries and occur more frequently in Eastern/Asian countries [1–6]. These are mostly aggressive malignancies that are difficult to treat and have generally poor outcomes, especially when compared with B-cell lymphomas [1, 2, 5]. The majority of PTCL patients either never achieve initial remission or sustain relapsed-free disease. The 5-year event-free or progression-free survival rate is less than 20–30% in most series without obvious significant improvement in the modern era [2]. There remains a critical need to learn more about the biology of this disease and to discover new and novel therapies for PTCL patients.

An ideal animal model to study human cancer should possess comparable histopathological features, biological behavior (e.g., organ distribution, metastases, etc.), and molecular and genetic characteristics [7]. The canine is a highly appealing model for cancer research due to these aforementioned similarities in addition to having a fully intact immune system, similar clinicopathologic features of the disease, a more comparable body size than the mouse model and pharmacokinetic properties. The relatively shorter lifespan of the canine allows lifelong follow-up over an abbreviated timeframe [8–10]. Furthermore, prior studies based on canine cancers such as canine osteosarcoma, prostate cancer, diffuse large B cell lymphoma (DLBCL), mucosal melanoma, and bladder cancer suggests close biological and molecular resemblances between canine and human cancers [8, 11].

Lymphoma is the most commonly reported hematopoietic tumor in the dog. Furthermore, the incidence of lymphoma in dogs occurs at a frequency of 2–5 times compared to humans with estimations that one of every fifteen dogs will develop lymphoma over their lifetime [8, 11]. Interestingly, approximately 30% of non-Hodgkin lymphomas (NHLs) in the canine represent the T cell lymphoma (TCL) subtype [8, 11]. In 2005, the canine genome was completely sequenced and a high degree of sequence similarity compared to human genome was identified [12]. There were 650 sequences in the dog (versus 146 sequences in mouse) that were aligned in comparison with the human. Furthermore, recent comparative (i.e., canine and human) genomic data have emerged in NHL, primarily in B-cell lymphoma [13–15].

The classification of lymphoma in the canine continues to evolve as standardization of different antibodies are emerging and immunophenotype is essential for appropriate diagnoses. The Kiel/REAL classifications incorporate morphologic, genotypic and immunophenotypic features to characterize canine lymphoma and apply the WHO classification. In the canine, the most frequent subtypes of T cell lymphoma are pleomorphic mixed and lymphoblastic [16]. The classification mirrors the overall aggressive nature of the disease that is seen in human TCL and the percentage of canine to human adult T-lymphomblastic lymphoma is similar (about 17% of T-NHLs). On the other hand, small clear cell (t-zone) lymphoma are described in the canine (e.g., in golden retrievers) without a correlate in humans and indolent lymphomas are typically not found in the canine [16].

Given the relatively nascent field of comparative oncology, and moreover, the paucity of comparative biologic data of PTCL in canine lymphoma, we sought to establish mutational congruence of the canine with known human PTCL mutations. We defined morphologic and immunohistochemistry (IHC) data in canine TCL (cTCL) cases, which was followed by a customized Human/Canine Cancer Hotspot Panel (hcCHP) with DNA sequencing isolated from canine TCL to determine prevalent mutations in cTCL. In addition, we identified potential tumor associated mutations that may enrich our biologic understanding of PTCL as well as lead to potential determination of rationale drug targets for canine and human clinical trials.

## RESULTS

### hcCHP panel identifies somatic and germline mutations in cTCL

**Figure 1:**
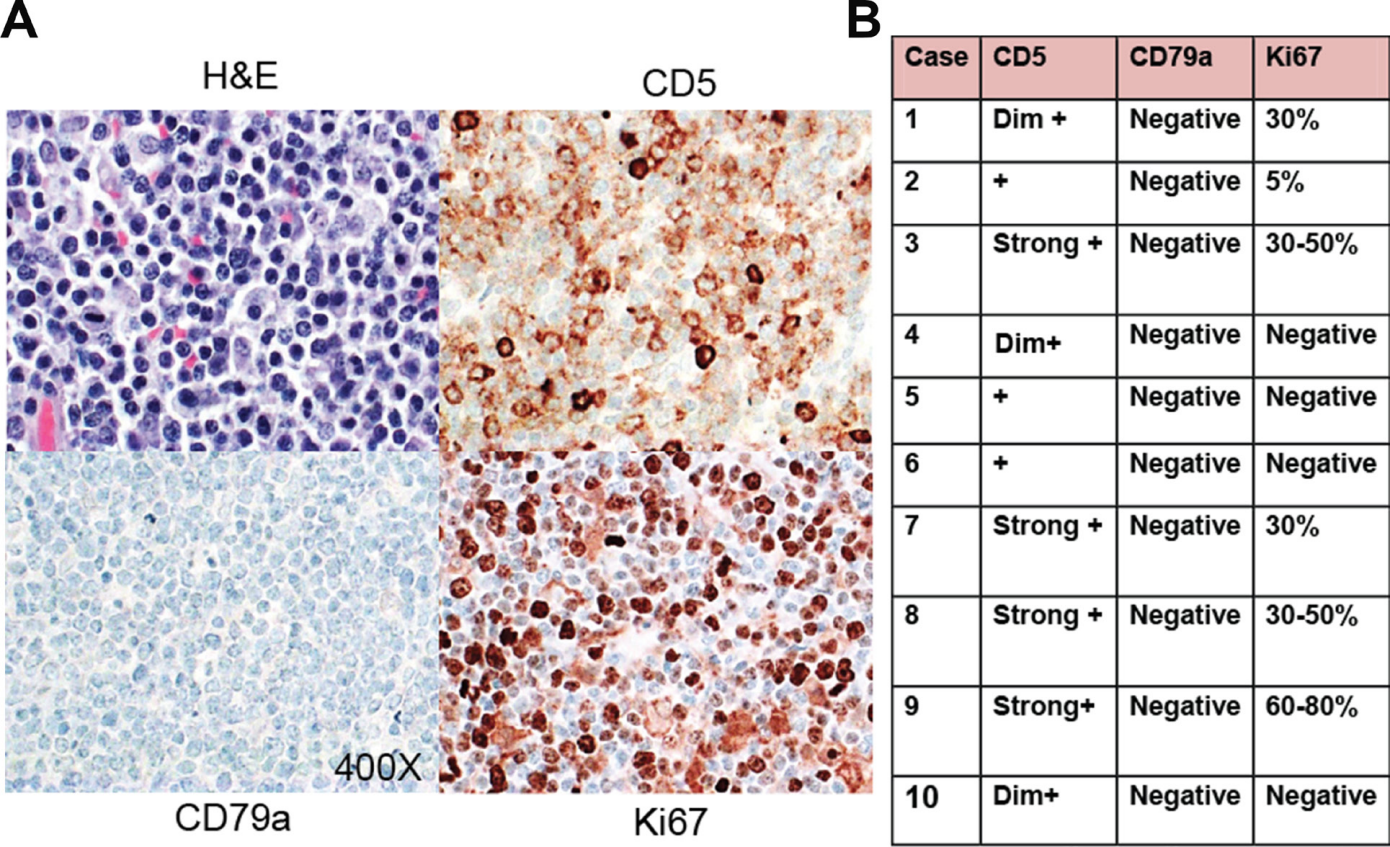
Immunohistochemistry of canine PTCL (**A**) Representative images (400X magnification) of paraffin embedded tissue stained with Hematoxylin & Eosin (H&E), positive cell surface staining for CD5, negative staining for CD79 and positive nuclear staining for Ki67, by immunohistochemistry in canine lymphoma tissues. (**B**) Summary table of results indicating the intensity of immunostaining in the individual specimens performed using canine lymphoma tissues.

Our panel of canine lymphomas included the canine breeds: 4 mixed breeds, 5 Golden Retrievers, 1 Boxer, 1 Poodle, 1 Yorkshire Terrier, 1 Bichone Frise, 2 German Shepards and 1 Rhodesian Ridgeback. The T cell origin of these canine lymphoma cases were confirmed by IHC based on T cell morphology; whereby 100% were CD79a (B cell marker) negative, 100% CD5+ (T cell marker), and there was variable Ki67 (marker of proliferation) with diffuse staining architecture (Figure 1), as has been reported in the literature. [16–18] Human anti-CD3, anti-CD4, anti-CD8, anti-CD30 were largely non-reactive in these primary PTCL canine samples.

For DNA sequencing of these cTCL cases, hcCHP was designed to include regions in 68 genes commonly mutated in human lymphoma that included genes representing key signaling pathways (e.g., NFκB and *TNFAIP3*), WNT/B-Catenin (e.g., *APC, CHD8, CELSR2*), and NOTCH (*NOTCH1, FBXW7*), epigenetic processes (e.g., *EP300, CREBBP, TET2, DNMT3A*), tumor suppressor pathways (e.g., *TP53, ATM, RB1, CUL9, PRKDC*) and p53-related genes (e.g., *TP53, TP63, CDKN2A, WWOX, and ANKRD11*). A complete list of genes included in this panel is included as Table 1.

**Table 1:** Canine cancer hotspot panel

**A. Derived from human hotspot panel**									
ABL1 (19)	AKT1 (6)	ALK (8)	APC (164)	ATM (24)	BRAF (77)	CDH1 (7)	CDKN2A (108)	CSF1R (8)	CTNNB1 (73)
EGFR (123)	ERBB2 (19)	ERBB4 (13)	EZH2 (11)	FBXW7 (25)	FGFR1 (2)	FGFR2 (8)	FGFR3 (17)	FLT3 (30)	GNA11 (5)
GNAQ (6)	GNAS (12)	HNF1A (10)	HRAS (26)	IDH1 (15)	IDH2 (12)	JAK2 (5)	JAK3 (6)	KDR (11)	KIT (139)
KRAS (63)	MET (18)	MLH1 (1)	MPL (0)	NOTCH1 (20)	NPM1 (28)	NRAS (35)	PDGFRA (26)	PIK3CA (97)	PTEN (146)
PTPN11 (28)	RB1 (18)	RET (17)	SMAD4 (31)	SMARCB1 (11)	SMO (5)	SRC (1)	STK11 (22)	TP53 (1087)	VHL (124)
**B. Additional genes**									
CCR4 (3)	CD28 (3)	CHEK2 (5)	CREBBP (9)	DNMT3A (6)	FAS (3)	FYN (3)	IL2RG (3)	IL7R (4)	JAK1 (12)
MYC (5)	PLCG1 (3)	PRDM1 (6)	RHOA (1)	STAT3 (6)	STAT5B (4)	TET2 (16)	TNFAIP3 (9)		

List of custom panel consisting 50 oncogenes and tumor suppressors designed to target 2,855 COSMIC mutations that occur frequently in human cancers selected for targeted sequencing of canine lymphoma specimens.

**Figure 2:**
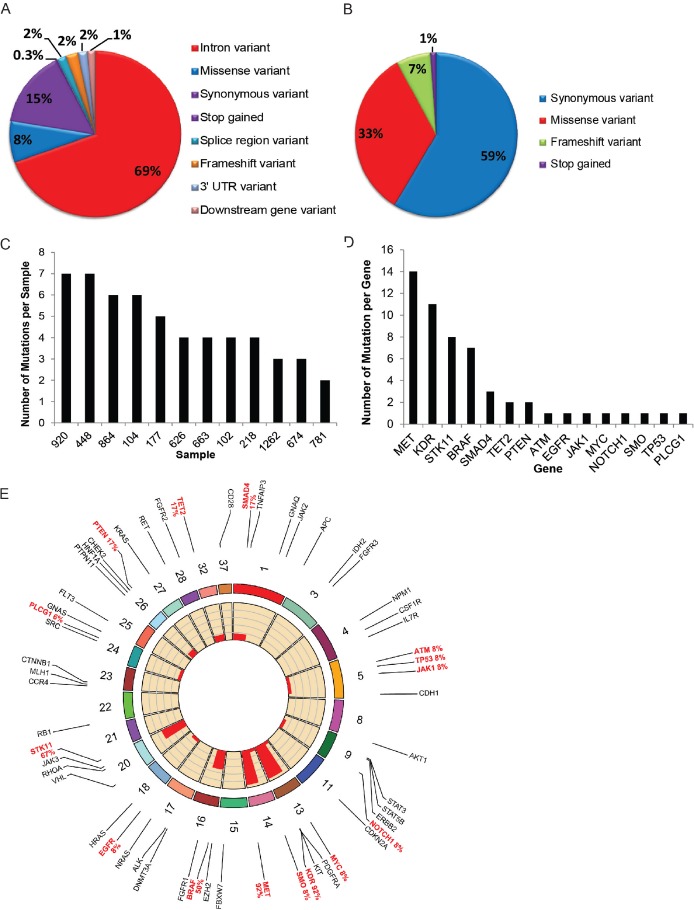
DNA sequencing (**A**, **B**) Pie chart represents the distribution of mutations within the entire gene or coding regions and all consequences. (**C**) Frequency chart show number of mutations (in y-axis) in individual canine T-cell lymphoma (cTCL) tumor represented by specimen number indicated in x-axis. (**D**) Frequency chart show number of mutations (in y-axis) plotted against individual gene represented in x-axis. (**E**) Frequency plot using RCircos represents frequency of somatic mutations in cTCL based on individual genes and corresponding chromosomal locations in the Canine Genome (some regions were excluded). Red text indicates genes with somatic mutations while black text indicates other gene regions that were sequenced but were without the presence of a somatic mutation.

DNA sequencing resulted in 4,527,638 total reads with an average length of 229 bases and 708× coverage per sample from two runs consisting of 16 tumor or normal samples where 12 of the 16 (75%) were matched normal and tumor pairs. The most prevalent consequence mutations were intron variations (69%), synonymous (15%), and missense variations (8%) (Figure 2A). Within the coding regions, 59% were synonymous variants, 33% missense mutations, 7% frame-shift, and 1% were stop/gain mutations (Figure 2B). These represented a total of 346 variants with the majority being represented as germline (258/346, 75%), 32 somatic variants, 8 missense mutations, one variant in a splice region, and the remaining were intron or synonymous variants (Supplementary Table 1). On average, a frequency of 4 mutations per sample was noted (Figure 2C) with occurrence of 7 mutations being highest and 2 mutations being the lowest. There were >7 mutations detected in the *MET, KDR, STK11* and *BRAF* genes; and <7 mutations detected among *SMAD4, TET2, PTEN, ATM, EGFR, JAK1, MYC, NOTCH1, SMO, TP53* and *PLCG1* genes (Figure 2D).

Among the somatic mutations detected in the MET gene (located on chromosome 14), two missense (C/G) variants were detected in 1/12 (8%) and 8/12 (67%) tumors. One synonymous mutation (T/C) was detected among 5/12 (42%) tumors, with 2 of these tumors showing overlap of both C/G and T/C mutations when examining tumors that had matched normal tissues. Other significant somatic mutations were detected on Chromosome 16; BRAF gene (42% tumors), Chromosome 13; KDR (83% tumors) and Chromosome 20; STK11 with one missense variant detected in 8 of 12 (67%) samples in the matched normal and tumor pairs. The Circos plot includes a detailed summary of both germline and somatic mutation consequences (Figure 2E).

### Activation of MAPK signaling in cTCL

Next, we sought to determine if these mutants or variants detected in cTCL were reflected in abnormal or aberrant protein expression. It should be highlighted that *MET, KDR* (VEGFR) *STK11* and *BRAF* are all upstream signaling components in the MAPK pathway [19–22] and aberrant MAPK signaling is a known oncogenic mechanism in lymphoma [23]. Somatic mutations and copy number alterations of these genes have been reported to activate MAPK and NFκB signaling pathways in human TCL [22] suggesting that the observed mutations, if functional, could result in activation of MAPK and NFκB signaling in cTCL. We also analyzed for MET expression and activation of MAPK signaling components to determine the extent of MET involvement in cTCL.

**Figure 3:**
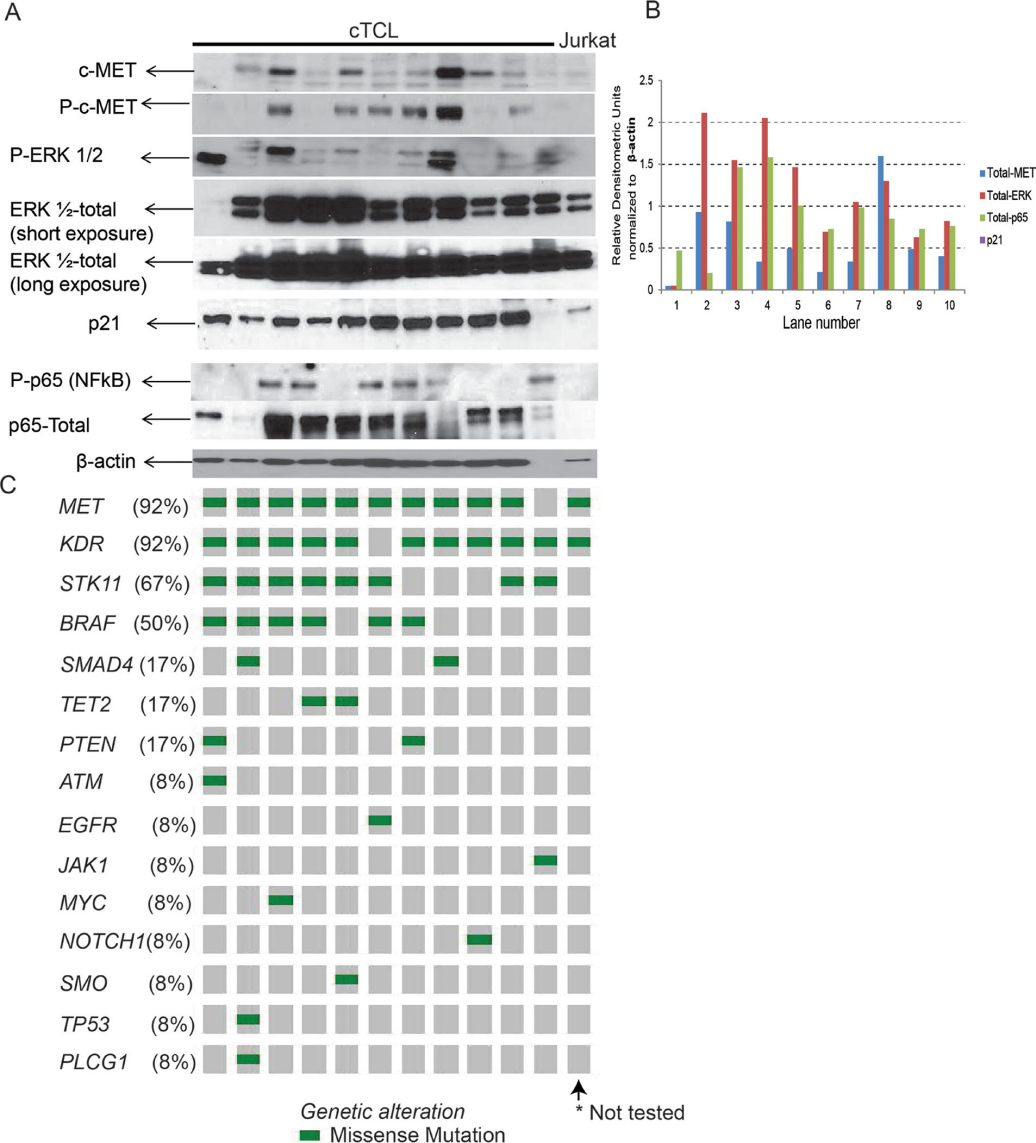
Protein analysis and mutational mapping SDS-PAGE and Western blot validation (**A**), and histogram (**B**) showing densitometry of Western blot protein expression of oncogenic signaling pathways in cTCL protein samples with mutant genes corresponding to individual cTCL represented in (**C**) as Oncoprint mapping of somatic mutations and frequency of mutations detected in these samples.

By Western blot analysis, we observed expression of the MET protein in 10/11 (91%) cTCL cases, increased phosphorylation of MET in 6/11 (55%) cTCLs, and mutations in MET were detected in 92% of cTCL tumors. Due to lack of appropriate antibodies to detect canine STK11, KDR or BRAF expression and also considering MAPK is a MET-dependent mechanism, we investigated ERK and NFκB activation and expression in cTCL as markers of oncogenic activation in response to the observed gene mutations. We observed increased phosphorylation of ERK ranging from mild to intense expression in all (100%) cTCL tumor samples (Figure 3A), while consistently high expression of ERK was noted in all cTCL cases. Similarly, 6/11 (55%) cTCL tumors showed increased phosphorylation of NFκB (p65), with all 11/11 (100%) cTCL tumors showing moderate to high levels of NFκB expression (Figure 3A). STK11 mutation is known to induce p21 expression predominantly in lung adenocarcinoma, [24] and our observation from the western blot analysis indicate high levels of p21 expression in 10/11 (91%) cTCL tumors (Figure 3A).

Comparing the expression levels by densitometry, normalized to β-actin expression, we observed that expression of MET and ERK were positively correlated (Correlation Coefficient = 0.467) (Figure 3B). Expression of NFκB or p21 were elevated along with MET in a tumor specific manner, but lacked overall correlation (Figure 3B) suggesting that MET-MAPK could potentially be a relevant mechanism in cTCL. Overall, we observed that expression of MAPK, NFκB and p21 pathway components were noted in most cTCL tumors which was expected based on the frequency of highly mutated (>7 mutations) genes for *MET* (92%)*, KDR* (92%)*, STK11* (67%) and *BRAF* (50%) (Figure 2D and Figure 3C).

Using Sanger sequencing, we next validated the most damaging mutations by filtering away variants with low quality and low allele frequencies and focusing on only variants found in tumors, but not in matched normal samples. This analysis revealed three variants of high quality. Among these were two variants found in 1 of 16 (6%) samples, MYC, a heterozygous missense mutation (chr13:25203051, c.185C>T, p.Ser62Phe) with frequency of 40.9%, interestingly, existence of this variant corresponded to the Canine genome location (CanFam3.1) - chr13:25203051-25203052 and in the human genome location (hg38) - chr8:127738447-127738448 has been previously reported in cutaneous squamous cell carcinoma [25], however, frequency for the existence of this variant is unknown. TP53, a heterozygous missense mutation (chr5:32563389, c.715G>A, p.Arg239Trp) with an allele frequency of 36.4% was identified. This variant is also known to exist in multiple human solid tumors corresponding to human chromosomal location chr17:7674218 [26], which is potentially implicated in oncogenic pathogenicity. The third variant found in 8 of 12 of the tumors samples (75%), but not in the matched normal samples was MET, a heterozygous missense mutation (chr14:55699186, c.3804C>G, p.Asp1268Glu) with low frequency (<=5%) in all but one sample (41%). Furthermore, by western blot analysis, we noted increased Myc protein expression in 2 cTCL tumors and phosphorylation of p53 protein in 4 cTCL tumors, (total protein was undetectable with our antibody). Despite the presence of this mutation in only one cTCL sample, increased p53 phosphorylation corresponded to a previously noted observation showing discordance between the frequency of *TP53* mutation and protein expression [27].

## DISCUSSION

Cancer in the canine (companion animal) is a naturally occurring disease in pets living in a shared environment alongside humans. Studies focusing on canine cancer cancers may provide biological insights beneficial both to humans and dogs. Lymphoma is the most commonly reported hematopoietic tumor in the dog. Incidence of lymphoma in dogs occurs at a frequency 3–5 times higher compared with humans (incidence 2.09 per 100,000 individuals) and it is estimated that approximately one of every fifteen dogs will develop lymphoma in their lifetime [8, 11]. Among canines lymphoma cases in Western world, approximately 30–40% are TCL with the remainder being B-cell lymphoma [8, 11]. This is compared with incidence of TCL in humans in the Western world of only 10–15% [11]. We undertook a comparative biologic study of known human PTCL genomic alterations to establish morphologic, IHC and DNA sequencing data from cTCL. We identified multiple somatic variants including TNFAIP3, ERBB2, MET, p53 and MYC that were confirmed on protein studies. To the best of our knowledge, these genomic analyses are the most comprehensive undertaking comparing cTCL with human TCL. In interpreting these observations, several factors should be considered.

The canine model is ideally suited for translational cancer research. Although murine experimental models are the cornerstone of preclinical oncology studies, most murine models lack innate immunity and have genetic dissimilarities that preclude reliable translation of murine biology into human clinical trials [9]. Oncogenic viruses, for example, are important to lymphomagenesis in humans but there are no large animal models aside from the dog that are affected by spontaneous viral infection (e.g., EBV disease). Viral particles have been detected in companion dogs and retroviral particles produced from sezary and LGL leukemia cell lines [28, 29].

Furthermore, specific dog breeds have distinct, significant and reproducible predisposition for developing certain lymphoma subtypes [8, 9, 30]. These breed specific incidences include TCL in 85% of Boxers, TCL almost exclusively in Asian and modern Spitz breeds, equal frequencies of TCL and BCL in Golden Retrievers, and 70% BCL and 30% TCL in mixed breeds [8, 11]. Breeding has selected for specific physical traits resulting in a finite genetic pool making it possible to study of genetic susceptibility to specific diseases. Additionally, frontline treatment for dogs with newly diagnosed cTCL closely mirrors that of humans with PTCL using cyclophosphamide, doxorubicin, vincristine and prednisone (CHOP) therapy. The outcomes for these treatments in both human and cTCL are sub-optimal. In humans, long-term disease-free survival for most subtypes of peripheral TCL are less than 30% [2], and for dogs with TCL, treatment is mainly palliative. Continued biologic understanding of TCL is critically needed and the identification of new and novel therapies is highly desired.

To date, the majority of studies in canine lymphoma have been focused on B-cell lymphoma (BCL) and these studies have resulted in parallel advances beneficial to both human and canine BCL patients [8, 14]. Comparative gene expression profiling identified common signaling pathways in human and dog BCL that let to identification of *NF*κ*B, PI3K* and *JAK/STAT* pathways as potential drug targets [13]. Prior studies based on exome sequencing in a limited number of cases showed that ERBB2 and PI3K were top canonical pathways frequently mutated in canine TCL [30]. Comparing this exome data to our targeted findings reported here resulted in 47% overlap in these previously discovered variants (Supplementary Table 3). Among primary cTCL cases sequenced in the current analyses, we found that *MET, KDR, STK11* and *BRAF* were the most frequent somatic mutations with *SMAD4, TET2, PTEN, ATM, EGFR, JAK1, MYC, NOTCH1, SMO, TP53* and *PLCG1* mutated less often.

It is important to note that *MET, KDR* (VEGFR), *STK11* and *BRAF* are upstream signaling components in the MAPK pathway [31–33]. To further interrogate MAPK signaling, we examined ERK (i.e., the only known direct downstream substrate of MEK). It has been previously that overexpression of MET is an oncogenic hallmark signature in human PTCL [34]. Moreover, in transgenic mice experimental model, MET expression resulted in development of thymic TCL with high penetrance [35], suggesting an important role for MET in the oncogenesis of TCL. We therefore examined protein expression with ERK and MET finding that 100% of primary cTCL cases had strong ERK phosphorylation and 91% of samples had MET constitutively stabilized. Furthermore, MET and ERK were strongly correlated. This suggests a larger role for therapy targeted at the MET receptor and/or combination therapies to its downstream targets. Multitargeted small molecule tyrosine kinase inhibitors of dual ALK and c-MET inhibition have been studied and FDA approved in non-small cell lung cancer and c-MET inhibition may be a potential therapeutic target in human lymphomas. PF-2341066, a small molecule inhibitor of c-MET and ALK, has been administered in ALCL with inhibition of tumor progression and apoptosis, as well as other c-MET inhibitors (e.g., SU11274 and PHA665752). These may be examined pre-clinically in the canine and other models to further our understanding of their effects [36].

TNFAIP3 (TNF Alpha Induced Protein 3) is an ubiqitin-editing enzyme that inhibits NFκB activation and TNF-mediated apoptosis, is a mediator of inflammatory function involving the T cell receptor (TCR) and is an activator of T lymphocyte function. Natural variants resulting from somatic mutation of this gene in humans has been reported to increase NFκB activation [37]. Several protein coding transcript variants of the TNFAIP3 gene reported in B-cell lymphomas are predominantly nonsense or frameshift mutations [38]. Activation of TCR signaling is reported as a pathogenic mechanism in human PTCL otherwise non-specified and prior studies have shown that this is associated with TNFAIP3 missense mutations in 11% of cases [2, 39]. We found here that 55% of cTCL tumors had phosphorylated NFκB (p65) expression with 100% of tumors showing moderate/high NFκB protein expression.

There are several limitations of the current series that should be acknowledged. We analyzed a total of 16 cTCL tumor tissues with histological diagnosis and used a “hotspot” Panel that covers regions in 50 oncogenes and tumor suppressors designed to target 2,855 COSMIC mutations occurring frequently in human cancers. It will be necessary to confirm the current results in larger numbers of cTCL cases. This may also help to identify the significance of variants that were germline as it is difficult to draw conclusions on their contributions here as the results may be reflective of differences in canine breed. Further, the DNA sequencing here was designed to include regions in 68 genes commonly mutated in human lymphoma that included genes representing key signaling pathways; a more comprehensive and exploratory sequencing investigation may be warranted to identify additional mutations not analyzed in the current study. Finally, the mutations identified here appeared to occur across all breeds (Supplementary Table 2), however the sample size here may have limited our ability to fully elucidate breed-specific characterization of genetic changes.

Taken together, the mutational spectrum and western blot analysis revealed a prominent overlap of human PTCL and cTCL at the molecular and protein level. DNA sequencing of the canine genome sheds light on the prevalent genomic mutations in TCL, namely the MAPK pathway and NFκB signaling. Further studies are warranted to confirm the mechanistic importance of the mutations identified here, however, MET, ERK, NFκB, and other MAPK substrates may serve as potential targets for the treatment of TCL. The canine may therefore be leveraged in the future as a unique tumor model to not only test potential drug candidates, but to also examine genomic and/or proteomic biomarkers of efficacy and outcome in TCL.

## MATERIALS AND METHODS

### Canine subjects

Tumors and normal tissues from canine subjects treated at Cummings School of Veterinary Medicine at Tufts University were obtained following IACUC, IRB approval and with consent from the pet owners. A total of 16 tumor tissues with histological diagnosis of TCL (14 fresh frozen tumors, 2 embedded in paraffin blocks) and 16 matched normal frozen samples stored in the repository were used for DNA and protein extraction. Primary lymph node tissue from dogs euthanized for medical reasons besides cancer were used as normal controls. The cell of origin (i.e., T cell vs B cell) was determined by morphology and IHC stains using human antibodies against CD3, CD5, CD8, CD30, CD79a and Ki67.

### DNA and protein extraction

Canine DNA was extracted using the QIAamp DNA Mini Kit (Qiagen, USA). Next generation sequencing was performed with the Ion Torrent Personal Genome Machine. Additional validation was performed with DNA extracted from matched normal samples from 12 of the original cTCL tissue and 4 distinct normal canine tissue samples. Protein lysates were prepared from primary cTCL tumor biopsies and human PTCL cell lines and analyzed by Western Blot for comparison and assessment of potential pathway/biomarker overlap, as described before [40]. Briefly, equal amounts of protein was loaded and run by electrophoresis using 4–15% Criterion precast gel (Biorad, USA), transferred to nitrocellulose membrane, blocked using 5% milk in TBS consisting 0.1% tween 20, followed by overnight incubation with primary antibodies (purchased from Cell Signaling Technology), washing and probing with appropriate HRP conjugated secondary antibodies and detection using Westfemto reagent (Pierce Biotech, USA).

### Human/canine cancer hotspot panel (hcCHP) design

A customized panel for targeted sequencing was informed by the COSMIC database and PubMed, and included a commercially available human Cancer “hotspot” Panel (hcCHPv2, ThermoFisher Scientific, USA). The latter covered regions in 50 oncogenes and tumor suppressors designed to target 2,855 COSMIC mutations that occur frequently in human cancers. Additionally, coordinate conversions between the hcCHPv2 and canFAM3 genomes [12] used liftOver from the UCSC Genome Browser Utilities [41] to identify and map common variants. Additional customization included another 101 regions in 18 genes that were added to this panel. The full panel is listed in Supplementary Table 1.

### Targeted next generation sequencing

Library preparation, templating and sequencing was performed as previously published [42]. Briefly, the Ion Ampliseq Library Kit 2.0 (Thermo Fischer Scientific) was used to for multiplexed PCR amplification from 20 ng of genomic DNA. Amplicons were then barcoded, quantified and pooled for sequencing on the Ion Personal Genome Machine (Thermo Fischer Scientific). Template-positive ISPs were loaded onto Ion 318v2 chips and sequenced using Ion PGM Hi-Q Sequencing Kit (Thermo Fischer Scientific) with 400 bp read lengths. The sequencing data was processed using Torrent Suite Software and Variant Caller v5.0.4.0. Sequencing results for called variants can be found in Supplementary Table 3.

## SUPPLEMENTARY MATERIALS TABLES








