# Correction: Comparative oncology DNA sequencing of canine T cell lymphoma via human hotspot panel

**DOI:** 10.18632/oncotarget.26114

**Published:** 2018-09-07

**Authors:** J. Tyson McDonald, Athena Kritharis, Afshin Beheshti, Monika Pilichowska, Kristine Burgess, Luisel Ricks-Santi, Elizabeth McNiel, Cheryl A. London, Dashnamoorthy Ravi, Andrew M. Evens

**Affiliations:** ^1^ Cancer Research Center, Hampton University, Hampton, VA, USA; ^2^ Division of Blood Disorders, Rutgers Cancer Institute of New Jersey, New Brunswick, NJ, USA; ^3^ WYLE, NASA Ames Research Center, Moffett Field, CA, USA; ^4^ Division of Pathology, Tufts Medical Center, Boston, MA, USA; ^5^ Cummings School of Veterinary Medicine, Tufts University, Boston, MA, USA

**This article has been corrected:** The correct author name is given below:

**Cheryl A. London**

Original article: Oncotarget. 2018; 9:22693-22702. https://doi.org/10.18632/oncotarget.25209

