# Supplementary material for: Comparative oncology DNA sequencing of canine T cell lymphoma via human hotspot panel
Source: Oncotarget. 2018 Apr 27;9(32):22693–702. doi: 10.18632/oncotarget.25209 (PMC5978258; doi:10.18632/oncotarget.25209)
Supplement: Supplementary file 1 [file oncotarget-09-22693-s001.pdf]

Comparative oncology DNA sequencing of canine T cell lymphoma via human hotspot panel

SUPPLEMENTARY MATERIALS

Supplementary Table 1: All mutations screened and identified. See Supplementary_Table_1

Supplementary Table 2: Mutations categorized based on canine breed. See Supplementary_Table_2

Supplementary Table 3: Targeted sequencing data for 346 called variants. See Supplementary_Table_3
